# Is spacing really the “friend of induction”?

**DOI:** 10.3389/fpsyg.2014.00259

**Published:** 2014-03-31

**Authors:** Peter P. J. L. Verkoeijen, Samantha Bouwmeester

**Affiliations:** Department of Psychology, Erasmus University RotterdamRotterdam, Netherlands

**Keywords:** replication, spacing effect, inductive learning, distributed practice, new statistics

## Abstract

Inductive learning takes place when people learn a new concept or category by observing a variety of exemplars. Kornell and Bjork (2008) asked participants to learn new painting styles either by presenting different paintings of the same artist consecutively (massed presentation) or by mixing paintings of different artists (spaced presentation). In their second experiment, Kornell and Bjork (2008) showed with a final style recognition test, that spacing resulted in better inductive learning than massing. Also, by using this style recognition test, they ruled out the possibility that spacing merely resulted in a better* memory* for the labels of the newly learned painting styles. The findings from Kornell and Bjork’s (2008) second experiment are important because they show that the benefit of spaced learning generalizes to complex learning tasks and outcomes, and that it is not confined to rote memory learning. However, the findings from Kornell and Bjork’s (2008) second experiment have never been replicated. In the present study we performed an exact and high-powered replication of Kornell and Bjork’s (2008) second experiment with a Web-based sample. Such a replication contributes to establish the reliability of the original finding and hence to more conclusive evidence of the spacing effect in inductive learning. The findings from the present replication attempt revealed a medium-sized advantage of spacing over massing in inductive learning, which was comparable to the original effect in the experiment by Kornell and Bjork (2008). Also, the 95% confidence intervals (CI) of the effect sizes from both experiments overlapped considerably. Hence, the findings from the present replication experiment and the original experiment clearly reinforce each other.

## INTRODUCTION

In Kornell and Bjork (2008) reported a study that investigated the effect of spacing on inductive learning, i.e., learning a new category by observing different instances from that category. In Experiment 1a, participants studied 6 different paintings of 12 different unknown artists under two learning schedules. In the massed/blocked condition, a block of six paintings consisted of six different paintings by the same artist. By contrast in the spaced/interleaved condition, each block of six paintings consisted of six different paintings by six different artists. After the learning phase, participants received a transfer test in which they saw new paintings by the 12 artists from the learning phase. Each test painting was accompanied by the names of these 12 artists, and participants had to select the artist who created the painting. The results of Experiment 1a demonstrated that test performance was better after spaced learning than after massed learning; a finding that was replicated in a between-subject design in Experiment 1b.

However, in the discussion of the results of Experiments 1a,b Kornell and Bjork (2008) pointed out that their findings might simply indicate that spaced learning made people remember the label associated with a painter better than massed learning rather than that it had led to a more effective induction of the artists’ styles. To rule out this alternative account, they replicated Experiment 1a with a final test better suited to measure inductive learning than the original *labeling *test. Specifically, in Experiment 2 participants took a recognition test consisting of new paintings by the previously learned artists and paintings by artists whose work was not presented during the learning phase. For each test painting, participants had to indicate whether it was by a familiar artist (i.e., an artist from the learning phase) or by an unfamiliar artists. Again, the results of this second experiment revealed that spacing led to better learning of the artists’ styles than massing.

The paper of Kornell and Bjork (2008) has had quite an impact as evidenced by the 54 citations to the paper since its publication (source: Web of Science, May 31, 2013). There are a number of reasons why the paper has been picked up by other scholars in the academic community. First of all, the finding that spacing leads to better inductive learning than massing is counterintuitive. In fact, based on theoretical and empirical arguments, Kornell and Bjork (2008) actually hypothesized a *massing *advantage in inductive learning. It was to their great surprise that the outcomes of their study pointed in the opposite direction. Secondly, Kornell and Bjork’s (2008) findings demonstrate that the positive effect of spacing is not confined to memory of *exact *repetitions. Instead, it also applies to more realistic learning conditions in which people learn to abstract a pattern from non-exact same-category repetitions and subsequently use this pattern in a transfer test. This in turn has important implications for educational practice because the benefit of spaced learning generalizes to complex learning tasks and outcomes, and not only to rote memory learning.

In the literature, several papers report successful conceptual and direct replications of Kornell and Bjork’s (2008) findings from Experiment 1a,b (e.g., Vlach et al., 2008; Kornell et al., 2010; Kang and Pashler, 2012; Zulkiply et al., 2012). However, to the best of our knowledge, there are no publications in which Experiment 2 from the Kornell and Bjork (2008) study was replicated. This is problematic because – like Kornell and Bjork (2008) suggest in their paper – the final test in their Experiment 2 is actually a better measure of induction than the final *labeling *test used in their Experiment 1a,b (and in the other papers in the literature for that matter). Given the impact the Kornell and Bjork (2008) paper has had so far, we think it is crucial to perform an exact replication of their Experiment 2. Such an exact replication would contribute to establish the reliability of the findings from Kornell and Bjork’s (2008) Experiment 2, and hence to more conclusive evidence of the spacing effect in inductive learning.

## MATERIALS AND METHODS

Note that the introduction and method section were preregistered. Therefore, the method section describes a planned procedure.

### SAMPLING PLAN

For the present study, we plan to recruit participants via Amazon Mechanical Turk (MTurk^1^). MTurk is an online system in which a *requester *can open an account and post a variety of research tasks. These tasks are referred to as *human intelligence tasks, *or *HITS. *People who register as MTurk *workers *can take part in HITS for a monetary reward. Simcox and Fiez (2014) list a number of advantages of the MTurk participants pool as compared to the (psychology) undergraduates participants pool from which samples are traditionally drawn in psychological research. First, MTurk participants are more diverse than undergraduates in terms of ethnicity, economic background and age and this benefits the external validity of MTurk research. In addition, contrary to many undergraduate participants’ pools, MTurk provides a large and stable pool of participants from which samples can be drawn year round. Third, experiments can be run very rapidly via MTurk. A disadvantage, however, is that the workers population might be more heterogeneous than the undergraduate population which generally leads to more within subject variance which in turn – ceteris paribus – deflates the effect-size. The reason why we decided to use MTurk participants in the present study is that we can collect a relatively large set of data (see the power calculation in the second part of this sub-section) within a short period of time.

Kornell and Bjork (2008) used undergraduate students instead of MTurk worker as participants of their original experiment. Hence, our sample is drawn from a different population than theirs. However, we think there are at least two arguments as to why this sample difference is not problematic. For one, nowhere in their original paper do Kornell and Bjork (2008) indicate that specific sample characteristics are required to obtain a spacing effect in inductive learning. Secondly, replicating the effect with a sample from a more heterogeneous population than the relatively homogeneous undergraduate population would constitute evidence for the robustness and generality of the spacing effect in inductive learning and, therefore, would rule out that the effect is restricted to a rather specific and narrow population.

We now turn to the power analysis for the present study. One requirement for proposals for the Frontiers special issue on “replications of important results in cognition” is that the power of planned studies, which should be calculated on the basis of the effect size of the existing/published evidence, should ideally be at least 0.95. The original experiment of Kornell and Bjork (2008) is the only experiment in the literature we are aware of that reports a spacing effect on inductive learning as measured with a yes/no recognition test. This experiment employed a within-subjects design with learning (massed vs. spaced) as independent variable and proportion of correctly recognized targets in the first quadrant of the yes/no recognition test (we will elaborate on this point later on in the confirmatory analysis plan) as dependent variable. The observed effect size (Cohen’s *d*) was 0.41.

However, for the present study, we will sample from a more heterogeneous population than Kornell and Bjork (2008) and this has consequences for the expected effect size and hence the power analysis. That is, given the larger variability, a larger difference in mean scores is required to obtain the same effect-size. Since we are not able to influence the raw mean difference, the expected effect-size is expected to be smaller in the MTurk population and a larger sample size is thus required to reach the threshold for statistical significance. The question then is how much larger the MTurk population standard deviation will be compared to the undergraduate population standard deviation. This question is hard to answer, but the findings from an unpublished study from our lab might be informative. That particular study included, amongst others, a retrieval practice/testing experiment (see Roediger and Karpicke, 2006 for more information about the testing effect) conducted in the psychological laboratory with undergraduate students and a direct replication conducted with MTurk workers. On a final memory test, a similar pattern of results was found in both experiments. However, more important for the present purposes is that the standard deviation of the MTurk results was about 1.25 times larger than the standard deviation of the undergraduate results^2^.

Although we cannot draw a general conclusion about MTurk/undergraduate ratio of standard deviations from a single comparison, the estimate of 1.25 is the best we have. Hence, for the present study, we assume that the standard deviation in MTurk population is 1.25 times larger than the standard deviation in the undergraduate population. In Kornell and Bjork’s (2008) original experiment the standard deviation of the difference scores was 0.24, implying that the expected standard deviation in the present experiment would be about 0.30. Hence, under the assumption that the mean spacing difference will be similar in both experiments, the expected effect size under the alternative hypothesis is approximately 0.1/0.30 = 0.33. With an effect size of 0.33, we need a sample of *n* = 120 for the desired power of 0.95 (for the calculation we used G^*^power, Faul et al., 2007). So, for the present experiment, we will test 120 MTurk workers.

### MATERIALS AND PROCEDURE

Dr. Kornell posted the stimulus materials employed in the Kornell and Bjork (2008) study on his website^3^. We used these materials (i.e., the original experiment’s materials) in the present experiment. The materials were 10 landscape or skyscape paintings by each of 12 impressionist artists (Georges Braque, Henri-Edmond Cross, Judy Hawkins, Philip Juras, Ryan Lewis, Marilyn Mylrea, Bruno Pessani, Ron Schlorff, Georges Seurat, Ciprian Stratulat, George Wexler, and Yie Mei). For each artist, six paintings were presented during the study phase of the experiment, and four paintings were presented as targets during the yes/no recognition test. Furthermore, for the yes/no recognition test, four distractor paintings were selected for each artist. These distractors items were also copied from the aforementioned website of Dr. Kornell, and as such they can be assumed to be identical to the distractors of the original experiment.

The procedure in the present experiment will be very similar to the procedure in the original experiment. That is, during the study phase, 72 paintings will be presented one by on a computer screen at a 3-s rate in 12 sets of 6 paintings. Six of these 12 sets will contain the paintings by a single artist (massed or blocked presentation, henceforth denoted as M) whereas the other six sets will contain six paintings by six different artists (spaced or interleaved presentation, henceforth denoted as S). The set order will be either MSSMMSSMMSSM or SMMSSMMSSMMS. Furthermore, within each of these presentation orders we will balance massed and spaced presentation over the artists. All in all this counterbalancing procedure will lead to four study sequences.

The recognition test will also be identical to the test in the original experiment. Specifically, the test will consist of four blocks of 24 paintings. Each block will contain 12 target paintings (new paintings of the 12 artists from the study phase) and 12 distractor paintings. The test will be self-paced and participants will receive no feedback on their response. After the test participants will be informed about the meaning of massed and spaced presentation. Subsequently, they will be asked to indicate which of the two presentation modes is most beneficial to learning. They will be given three options: “massed,” “spaced,” or “about the same.”

The exact instructions we will be using for the present experiment are presented below. These instructions were derived from Kornell and Bjork’s (2008; see pp. 586, 587, and 589) method section although we should mention that Kornell and Bjork did not provide the specific experiment instructions in their paper. The present experiment will be placed as a HIT on the MTurk website. This HIT will be accompanied by a specification of the reward for taking part in the experiment (i.e., 1 dollar) and the following short task description: “*This task consists of two phases. In the first phase you learn lists of paintings, in the second phase you have to decide whether paintings were familiar to you or not. The entire task will take about 15 min.*” MTurk workers, who accept the HIT will be asked to report their gender, date of birth, and their level of education. Also, they will be ask to indicate (either “yes” or “no”) whether they are native speaker of English. Subsequently, the will be taken to a next screen on which they will read the instructions for the study phase. The literal instruction will be:

“*In this experiment, you are going to learn the styles of 12 different artists by viewing 6 different paintings by each artist. So, you will see 72 paintings in total. These paintings will be presented automatically and one-by-one at a 3-s rate. Below each painting, the name of the artist will be presented. Furthermore, the paintings will be presented in blocks of 6. You will notice that some blocks contain the paintings by a single artist, whereas other blocks contain six paintings by six different artists.*

After the learning phase, you will receive a style test. This test consists of NEW paintings by artists whose paintings have been presented during the learning phase, and paintings by artists whose paintings HAVE NOT been presented during the learning phase. Your task will be to categorize a test painting as by a familiar artist or an unfamiliar artist.

*Click on* ≫* to start the learning phase.*”

After the study/learning phase, participants will perform a distractor task. The literal instruction will be:

“*The test phase of this experiment will start after the following distractor task: count backward by 3 s from 547 during 15 s. Please type in the answers in the textbox.*”

After the distractor task, participants will be taken to a next screen for the test phase. The literal instruction will be:

“The test consists of a number of paintings. These paintings will be presented in four blocks. Some of these paintings are NEW paintings by artists whose paintings have been presented during the learning phase, whereas other paintings are by artists whose paintings HAVE NOT been presented during the learning phase.

During each trial of the test phase, a painting will be presented with two buttons on the computer screen; one button is labeled “familiar artist,” and one is labeled “unfamiliar artist.” You have to select the “familiar artist” button if you think the painting is by an artist whose paintings had been presented during the study phase, and to select the “unfamiliar artist” button if you think the painting is by an artist whose paintings had not been presented during the study. If you do not know the answer, you have to make a guess. After you have made your decision, a next painting will appear. No feedback will be given during the test.”

Following the test phase, participants will we taken to a next screen for a final question. The experiment ends when an answer to this question has been provided. The literal instruction for the final question will be:

*“During the learning phase of this experiment, some blocks of six paintings were all by the same artist. This method of presentation is called “massed practice.” By contrast, other blocks contain six paintings by six different artists. This method of presentation is called “spaced practice.” Which method of presentation do you think helped you learn more, massed or spaced?’ Please select one of the following options: “massed,” “about the same,” or “spaced.”*”

### KNOWN DIFFERENCES FROM ORIGINAL STUDY

The present experiment will be a close to direct replication of Kornell and Bjork’s (2008) original experiment. There are, however, two differences between the two experiments. First, we will draw a sample from a different population than Kornell and Bjork (2008) did. Yet, we already explained under the *sampling plan *header why this difference is unlikely to be relevant. To briefly reiterate our arguments: (a) from Kornell and Bjork’s (2008) original paper it does not follow that specific sample characteristics are required to find a spacing effect in inductive learning, and (b) replicating the results with a sample from a different population would speak to the robustness and the generality of the spacing effect in inductive learning.

Second, Kornell and Bjork (2008) only employed a MSSMMSSMMSSM-order for the presentation of the six-paintings sets during the study phase. To counterbalance the artists across the massing and the spacing condition the artists were randomly assigned to the massing and spacing condition per participant. By contrast, we used both an MSSMMSSMMSSM presentation order and an SMMSSMMSSMMS presentation order, and within each presentation order we created two versions by counterbalancing the artists across the massing and the spacing condition. However, we do not see any theoretical and/or practical reason as to why this difference between our experiment and the original experiment should produce different outcomes.

### CONFIRMATORY ANALYSIS PLAN

For the present experiment, we will collect per participant – besides the previously mentioned demographic characteristics – the following test data: the proportion of correctly recognized massed targets, the proportion of correctly spaced targets, and the proportion of distractors incorrectly classified as “old,” i.e., the number of false alarms. We will collect these data per block of the recognition test. In addition, we will collect the participants’ responses to the final question. Prior to statistical analysis of the test data, we will execute a data cleaning plan. That is, we will exclude the data of a participant when a participant reports that he/she is not a native speaker of English.

Subsequently, and following the analysis procedure of Kornell and Bjork (2008), we will only analyze the correctly recognized massed and spaced targets from the *first block *of the recognition test. Kornell and Bjork (2008) conducted a repeated measures analysis of variance (ANOVA) on the proportion of correctly recognized targets with learning type (massed vs. spaced) as independent variable. We will conduct exactly the same analysis.

On the basis of the outcomes we will evaluate whether the replication attempt is successful. The evaluation will be based on the observed *p-*value, i.e., whether the observed effect is significant, the direction of the effect, the difference between the massed and the spaced means, and the standard deviation of the difference scores. Regarding the difference between the massed and spaced means, we expect to observe a spacing advantage that is similar (i.e., 0.1) to the one Kornell and Bjork (2008) found. Furthermore, and as pointed out before, due to the sample of MTurk workers we expect to find a standard deviation of the difference scores that is about 1.25 times larger than the standard deviation in Kornell and Bjork’s (2008) original experiment. All in all, we therefore expect to find a spacing effect in inductive learning with an effect size of about 0.33 (see the previous power analysis for the calculation of this expected effect size).

To determine whether the replication attempt has been successful, we plan to use the criteria recently put forward by Simonsohn (unpublished paper)^4^. In this paper, Simonsohn proposes to evaluate replication attempts on the basis of a method that combines the *p-*value and the effect size of a replication attempt. With respect to the latter point, Simonsohn argues that it is important to assess on the basis of the outcome of a replication attempt whether the estimate of the population effect size is at least equal to a certain minimal value. What the minimal value should be is a subjective question, and the answer depends on the research being conducted. However, to provide a guideline, Simonsohn suggest (and he admits that this suggestion is fairly arbitrary) to use as the minimal effect size, the effect size associated with a power of 0.33 for the sample size of the *original study*. This minimum level is denoted as *d*_33%_. To evaluate the outcome of a replication attempt, the 95% CI of the effect size should be determined for the replication attempt. Subsequently, a replication attempt is only deemed successful if the observed finding is significant (i.e., the CI does not include 0) and if it is reasonable to assume that the population effect size is equal to or larger than the *d*_33%_ standard.

For the present study we will evaluate the outcome of the replication attempt on the basis of the above described decision procedure using 0.17, i.e., the *d*_33%_ of the original experiment by Kornell and Bjork (2008) as a threshold for a minimally required effect size.

## RESULTS

The results of the present experiment were obtained by exactly executing the sampling plan and procedure described in the “Methods” section above. Subsequently, we will present the outcomes of the present experiment starting with a description of the MTurk sample.

### SAMPLE

A total of 143 MT workers accepted the HIT. The experiment took 30 min and participants who finished the experiment received a payment of 1 dollar provided that they had taken the hit only once. The data of 12 MTurk workers could not be included in the data analysis because they either failed to complete the experiment or because they did the experiment twice. In addition, consistent with our analysis plan we excluded the data of another 8 MTurk workers from the data analysis because they indicated they were not native speakers of English. It should be noted that the later subset of 8 MTurk workers were paid for their participation. Due to these exclusions, the data analysis was performed on the data of the remaining 123 MTurk workers: this sample size meets the standard of 120 participants we set in our a-priori power analysis. The final sample consisted of 78 women (about 63%) and 44 males (about 36%); one MTurk worker (1%) chose to withhold gender information. Furthermore, with respect to highest level of education two MTurk workers (about 2%) reported less than high school, 14 MTurk workers (about 11%) reported high school/GED, 35 MTurk workers (about 28%) reported some college, 16 MTurk workers (about 13%) reported a 2-year college degree, 48 MTurk workers (about 39%) reported a 4-year college degree, 7 MTurk workers (about 6%) reported a Master’s degree, and one MTurk worker (about 1%) reported a professional degree (i.e., JD/MD). The mean age in years within the sample was 37 (range 19–73, *Median* = 34, *SD *= 12.26).

### CONFIRMATORY ANALYSIS

For all the analyses in this paper a *p-*value of 0.05 was used as a threshold for statistical significance. Also, for a measure of effect size we used Cohen’s *d. *Kornell and Bjork (2008) appeared to have calculated (they do not mention this explicitly) Cohen’s *d *for the crucial spacing effect comparisons by means of dividing the mean difference between the spaced and the massed condition by the standard deviation of the difference scores. Therefore, we will calculate Cohen’s *d *in the same manner. Following our analysis plan – and Kornell and Bjork’s (2008) analysis procedure for that matter – we analyzed the proportion of correctly recognized massed and spaced targets (i.e., the hit rate) from the *first block *of the recognition test. In our experiment, the mean hit rate for spaced items in that block was higher (*M* = 0.75, *SD* = 0.21) than for massed items (*M* = 0.66, *SD* = 0.22), *t*(122) = 4.180, *p* = 0.00005, *d* = 0.37. The mean false alarm rate was respectively 0.50 (*SD* = 0.21) for distractors coupled with massed items and 0.48 (*SD* = 0.22) for distractors coupled with spaced items, *t*(122) = 0.982, *p* = 0.328, *d* = 0.09.

After the experiment, participants were asked which of the two presentation modes were most beneficial to learning. Eight-seven participants (71%) reported that massed presentation resulted in most learning, 21 participants (17%) reported that both presentation modes were equally effective, and 15 participants (12%) reported that spacing had resulted in a better final test performance than massing. These metacognitive judgments are strikingly at variance with the spacing effect shown on the final test.

Kornell and Bjork (2008) reported a mean hit rate for spaced items of 0.77 (*SD* = 0.22) and of 0.67 (*SD* = 0.24) with an effect size of *d* = 0.41. The mean false alarm rates and the standard deviations were presented by means of bars in a figure and therefore it is not possible to give exact values. It seems that the mean false alarm rate in their experiment was about 0.50 for both spaced and massed distractors. Also, the mean false alarm rate did not differ significantly between spaced and massed distractors. In addition, the vast majority of the participants in Kornell and Bjork’s (2008) experiment (i.e., 80%) reported that massing had led to better learning than spacing. When we compare our replication experiment to the original experiment by Kornell and Bjork (2008) it becomes clear that the critical results are very much comparable.

In order to “formally” evaluate the replication attempt we calculated the 95% CI of the effect size, Cohen’s *d*, using Cumming’s (2012) exploratory software for confidence intervals (ESCI). Because *d *has a non-central *t-*distribution, CI for the corresponding parameter δ cannot be obtained from a formula. Instead, ESCI estimates the lower bound and the upper bound through an iterative approximations method. In the present study, this method resulted in a 95% CI of the effect size with a lower bound of 0.18 and an upper bound 0.55. According to Simonsohn’s (2014) criterion a replication attempt is successful if it rejects the null hypothesis (i.e., if the CI does not include the value of 0) and if it is reasonable to assume that the population effect size is equal to or larger than the *d*_33%_ standard. The *d*_33%_ of the original experiment of Kornell and Bjork (2008) is approximately equal to 0.17. In our replication attempt we found a significant spacing advantage and the CI of the effect size suggests that the population effect size is likely to be larger than the *d*_33%_ standard of 0.17. So, we think it is fair to interpret the results of the present experiment as a successful replication of Kornell and Bjork’s (2008) original experiment. This interpretation is also backed up by the similarity between the two experiments in terms of mean overall hit rate, the mean overall false alarm rate, the metacognitive judgments about the best presentation mode, as well as the effect size of the spacing effect for hit rates and false alarms.

Furthermore, following Cumming (2012) we calculated the 95% CI of the effect size for Kornell and Bjork’s (2008) experiment. The boundaries of this CI are, respectively, [0.18; 0.64]. This interval largely overlaps with the 95% CI of the present experiment [0.18; 0.55]. In sum, both experiments show a mean advantage of spacing over massing in inductive learning and the effect size of the spacing advantage is comparable between the two experiments. In addition, the 95% CI-s of the effect size overlap considerably. All in all, this strongly suggests that the outcomes of the two experiments should be considered as consistent. In fact, they even seem to reinforce each other and hence it is informative to combine both experimental results in a small scale random effects meta-analysis. The outcomes of this analysis revealed a combined effect size of 0.39 with a 95% CI of [0.24; 0.53]. The combined 95% CI is narrower than each of the individual 95% CI-s. This means that by combining both results we have obtained a more precise estimate of the spacing effect parameter in inductive learning.

### EXPLORATORY ANALYSIS

In this section, we will present the outcomes of four exploratory analyses suggested by an anonymous colleague who reviewed the proposal that gave rise to the present experiment. However, because statistical significance has no evidential value in exploratory research (see for instance De Groot, 2014), we will only provide relevant descriptive statistics. These findings may in turn inspire researchers to formulate and subsequently test new hypotheses about the mechanisms underlying the spacing effect in inductive learning.

We start with the analyses of the hit rates and false alarm scores in the four blocks of the final test (see **Tables 1** and **2**). Furthermore, **Table 3** presents *d*-prime as a function of test block and item type. The *d*-prime value for a given block and a given item type is based on the formula *Z*(mean hit rate) - *Z*(mean false alarm rate). We obtained the *d*-prime values in **Table 3** by entering the mean hit rate and the mean false alarm rate per block and per item type in the following online calculator^5^. The results in **Table 1** show that the advantage of spacing over massing varies over the blocks of the final test with positive spacing effects in block 1 and 3 and negative spacing effects in block 2 and 4. Moving to the false alarms in **Table 2**, we see that the overall mean tends to increase while moving from the first to the fourth test block. Kornell and Bjork (2008) report a similar decrease in recognition accuracy over test blocks. According to Kornell and Bjork (2008) this is due to the fact that trials during a recognition test may serve as learning events. Hence, when a participant in the first block of the test incorrectly classifies a distractor painting as old, characteristics of the new style may be added to the representation of familiar styles developed during the learning phase. As a result, the mental representation of old (i.e., presented during the learning phase) artists’ styles becomes contaminated with new styles from the distractors in the test phase, which in turns leads to an incline in recognition accuracy over the test blocks.

**Table 1:** Mean proportion correctly identified targets (hit rate) on the final test and standard deviation as a function of block and item type.

Block	Item type	Mean	SD
1	Massed	0.66	0.22
	Spaced	0.75	0.21
2	Massed	0.68	0.24
	Spaced	0.62	0.24
3	Massed	0.62	0.22
	Spaced	0.68	0.24
4	Massed	0.66	0.23
	Spaced	0.61	0.23

**Table 2:** Mean proportion of false alarms (distractors incorrectly identified as old) on the final test and standard deviation as a function of block and item type.

Block	Item type	Mean	SD
1	Massed	0.50	0.21
	Spaced	0.48	0.22
2	Massed	0.49	0.24
	Spaced	0.57	0.20
3	Massed	0.52	0.22
	Spaced	0.53	0.23
4	Massed	0.50	0.27
	Spaced	0.56	0.22

**Table 3:** *d*-prime on the final test as a function of block and item type.

Block	Item type	*d*-prime
1	Massed	0.41
	Spaced	0.73
2	Massed	0.52
	Spaced	0.13
3	Massed	0.26
	Spaced	0.39
4	Massed	0.41
	Spaced	0.13

Second, we investigated the correlation between the spacing advantage on targets in the first test block and age (in years). We found that the spacing effect tended to increase somewhat with age, *r *= -0.173 (this corresponds with an *R*^2^ of 0.03).

Third, we analyzed the median response times for spaced targets on the final test and massed targets. In Qualtrics these median reaction times can be calculated from the “first clicks” or the “second clicks.” The first click refers to the first keyboard action in a test slide performed by the participant. The first click could refer to a participant entering the response but it might also refer to a different action, such as placing the cursor in the response field. The second click always refers to the submission of a response to a test trial. Here, we report mean median reaction times for both types of clicks. The mean median first click response time was lower for spaced targets than for massed targets (*MD* = 0.076 s, *SD* = 0.47). A similar pattern was found for second clicks (*MD* = 0.074 s, *SD *= 0.37). Thus, and as expected from a common-sense line of reasoning, participants needed less time to respond to targets that were better learned (as indicated by the memory performance scores).

Fourth, we calculated the mean spacing advantage in the first test block for each of the four counterbalancing sequences. The mean spacing advantages (and the standard deviations of the difference scores) were, respectively: first sequence = 0.075 (*SD* = 0.26), second sequence = 0.046 (*SD* = 0.23), third sequence = 0.083 (*SD* = 0.24), and the fourth sequence = 0.19 (*SD* = 0.25). Hence, the mean spacing advantage appeared to differ somewhat between the four counterbalancing sequences.

## DISCUSSION

The present replication attempt was motivated by concerns about the validity of the final tests commonly used in the emerging field of research on the spacing effect in inductive learning (see Toppino and Gerbier, 2014 for an excellent recent review). Most studies in this field require participants to learn novel categories, such as new bird species or styles of unfamiliar painters. During the learning phase, a new instance from a category is presented along with the category label. Subsequently, on the final test participants are presented with *new* instances from the previously learned categories and they have to provide a correct label for each of them. However, it could be argued that this task measures participants’ memory for category labels rather than the induction of category specific characteristics. To provide a more valid measure of induction, Kornell and Bjork (2008) used a style/recognition test in their second experiment. The results on this test revealed a clear spacing effect: targets were more often judged as being familiar when learned through spaced/blocked presentation than when learned through massed presentation. Yet to the best of our knowledge, Kornell and Bjork’s (2008) second experiment has never been replicated. Hence, replication studies, such as the present study, are needed to obtain a more accurate estimate of the spacing effect in this kind of tests.

In the present study, the means and standard deviations on the final test target performance and distractor performance strongly resembled those in Kornell and Bjork’s (2008) study. Also, and in line with Kornell and Bjork’s (2008) results, we found a medium-sized spacing effect on the recognition/style test. Furthermore, our participants – like the participants of Kornell and Bjork (2008) – demonstrated rather poor metacognitive judgments. That is, although the final test demonstrated a clear spacing effect, the vast majority of our participants actually thought that massed presentation rather than spaced/blocked presentation resulted in the best final test performance. Hence, our results clearly buttress those of Kornell and Bjork (2008) and taken together they suggest that spacing is indeed beneficial in inductive learning.

A number of methodological recommendations follow from the present study. We found a spacing effect with a better (in terms of validity) measure of inductive learning than the commonly used labeling test. Therefore, future studies should use the recognition/style test – or a conceptually similar test – to assess participants’ inductive learning. However, we do not recommend the use of multiple test blocks. Since the present study was set up as a direct replication, we had to follow the exact procedure of the original experiment. This procedure involved the presentation of four blocks at the final test despite the fact that Kornell and Bjork (2008) already pointed out that the last three blocks should not be taking into consideration in the data analysis. Thus, when researchers plan to measure inductive reasoning with a recognition/style test, they are advised to limit the final test to a single block.

The data in the present study were obtained from a web-based sample of MTurk workers. Nevertheless, their mean performance, the mean spacing advantage as well as the variance in their performance were in line with Kornell and Bjork’s (2008) data, which were from a more traditional college student sample. Recently, other studies have also shown that data from web-based samples, consisting of unsupervised and completely anonymous participants, can yield data comparable to those collected in the psychological laboratory (e.g., Germine et al., 2012; Zwaan and Pecher, 2012; Birnbaum et al., 2013; Crump et al., 2013; Goodman et al., 2013). Moreover, web based samples have some clear advantages over lab samples. For example, web-based experiments allow for a fast collection of a large number of data. In addition, as compared to laboratory experiments with undergraduate students, web-based experiments involve demographically more diverse samples, enabling amongst others a broader generalizability of the results. Therefore, we think it is fair to say that web-based testing is likely to evolve into a valuable tool for conducting (cognitive) psychological research.

A final point we will address is the replication-evaluation approach put forward by Simonsohn (2014). Simonsohn (2014) proposes to combine statistical significance and effect-size estimation to evaluate results from replication attempts. More specifically, in Simonsohn’s (2014) approach two crucial questions are addressed with respect to a replication attempt: (1) is there a statistically significant effect, and (2) is it reasonable to assume that there is at least a small effect, with a small effect being defined as the Cohen’s *d *value associated with a 0.33 power of the original study (i.e., *d*_33%_). Although this approach is to be preferred above approaches focusing on either statistical significance or effect size, we think it has some limitations. First, it ignores relevant information from the original study because the *d*_33%_ value is entirely determined by the original study’s sample size. In our view, it would be better to take the original study’s effect-size estimation into account as well as the 95% CI of the effect size. To evaluate a replication attempt, the original 95% CI and the replication 95% CI should be compared on estimation precision, magnitude of the effect, significance and overlap between the CI-s. Such evaluation approach might strike readers as being too subjective. However, these readers should keep in mind that Simonsohn (2014) approach rests largely on an at least in part arbitrarily chosen – instead of objectively determined – *d*_33%_ value. Furthermore, despite a larger degree of subjectivity, the evaluation approach we propose might actually provide a more accurate description of the findings from the original experiment and its replication together. The latter aspect brings us to a second limitation of Simonsohn (2014) approach. As Simonsohn (2014) acknowledges in his paper, his evaluation approach only indicates whether a replication attempt should be considered as inconclusive, a success or a failure. This might sometimes be relevant, but considering the (ideally) cumulative nature of science, we think it is often more important to ask the question what the findings from a replication study add to the existing knowledge about the magnitude and the variability of an effect under interest (cf. Cumming, 2012). So, rather than competitively comparing the findings from a replication attempt and the original study, it would be more informative to combine them in a meta-analytical manner.

## CONCLUSION

The findings from the present replication attempt revealed a medium-sized advantage of spacing over massing in inductive learning comparable to the original effect found by Kornell and Bjork (2008). Also, the 95% CI-s of the effect size from the present experiment and the original experiment overlapped considerably. Thus, the findings from both experiments clearly reinforce each other and as a result the combined estimate of the effect size is more accurate than each of the effect-size estimates from the individual experiments alone. That said, the 95% CI of the combined effect is still wide, i.e., [0.24; 0.53], so more research is needed to obtain a more precise estimate of the spacing advantage in inductive learning as measured with a style/recognition test.

## Conflict of Interest Statement

The authors declare that the research was conducted in the absence of any commercial or financial relationships that could be construed as a potential conflict of interest. The Guest Associate Editor Rene Zeelenberg declares that, despite being affiliated to the same institution as authors Peter P. J. L.Verkoeijen and Samantha Bouwmeester, the review process was handled objectively and no conflict of interest exists.

## Footnotes

1 http://www.mturk.com

2 It should be noted that the estimation of the standard deviations ratio (MTurk/undergraduate), as derived from our unpublished study and the Roediger and Karpicke (2006) study, is based on standard deviations because both studies involved entirely between-subject designs. However, in a within-subject design, such as the replication study we are proposing, observations are correlated and therefore a larger standard deviation for MTurk participants versus undergraduate students does not imply a larger standard deviation of difference scores. Therefore, we are probably overestimating the standard deviation of the difference scores in the MTurk population. This in turn, will results in a higher power than the one we will compute subsequently.

3 http://sites.williams.edu/nk2/stimuli/

4 http://papers.ssrn.com/sol3/papers.cfm?abstract_id = 2259879

5 http://memory.psych.mun.ca/models/dprime/
